# Agricultural management practices influence the soil enzyme activity and bacterial community structure in tea plantations

**DOI:** 10.1186/s40529-021-00314-9

**Published:** 2021-05-18

**Authors:** Yu-Pei Chen, Chia-Fang Tsai, P. D. Rekha, Sudeep D. Ghate, Hsi-Yuan Huang, Yi-Han Hsu, Li-Ling Liaw, Chiu-Chung Young

**Affiliations:** 1 Department of Public Health and Medical Technology, Xiamen Medical College, Xiamen, 361023 Fujian China; 2 Engineering Research Center of Natural Cosmeceuticals College of Fujian Province, Xiamen Medical College, Xiamen, 361023 Fujian China; 3 Department of Soil and Environmental Sciences, National Chung Hsing University, Taichung, 40227 Taiwan; 4 Yenepoya Research Centre, Yenepoya University, Mangalore, Karnataka India; 5 School of Life and Health Sciences and Warshel Institute for Computational Biology, Chinese University of Hong Kong, Shenzhen, 518172 Guangdong, China; 6 Food Industry Research and Development Institute, Bioresource Collection and Research Center, HsinChu, 300 Taiwan; 7 Innovation and Development Center of Sustainable Agriculture, National Chung Hsing University, Taichung, 40227 Taiwan

**Keywords:** Agricultural management, Arylsulfatase, Bacterial richness, Bacterial diversity, Next-generation sequencing, Temporal change, Soil health

## Abstract

**Background:**

The soil quality and health of the tea plantations are dependent on agriculture management practices, and long-term chemical fertilizer use is implicated in soil decline. Hence, several sustainable practices are used to improve and maintain the soil quality. Here, in this study, changes in soil properties, enzymatic activity, and dysbiosis in bacterial community composition were compared using three agricultural management practices, namely conventional (CA), sustainable (SA), and transformational agriculture (TA) in the tea plantation during 2016 and 2017 period. Soil samples at two-months intervals were collected and analyzed.

**Results:**

The results of the enzyme activities revealed that acid phosphatase, arylsulfatase, β-glucosidase, and urease activities differed considerably among the soils representing the three management practices. Combining the redundancy and multiple regression analysis, the change in the arylsulfatase activity was explained by soil pH as a significant predictor in the SA soils. The soil bacterial community was predominated by the phyla Proteobacteria, Acidobacteria, Actinobacteria, Chloroflexi, and Bacteroidetes in the soil throughout the sampling period. Higher Alpha diversity scores indicated increased bacterial abundance and diversity in the SA soils. A significant relationship between bacterial richness indices (SOBS, Chao and ACE) and soil pH, K and, P was observed in the SA soils. The diversity indices namely Shannon and Simpson also showed variations, suggesting the shift in the diversity of less abundant and more common species. Furthermore, the agricultural management practices, soil pH fluctuation, and the extractable elements had a greater influence on bacterial structure than that of temporal change.

**Conclusions:**

Based on the cross-over analysis of the bacterial composition, enzymatic activity, and soil properties, the relationship between bacterial composition and biologically-driven ecological processes can be identified as indicators of sustainability for the tea plantation.

**Supplementary Information:**

The online version contains supplementary material available at 10.1186/s40529-021-00314-9.

## Background

Soil microorganisms play a key role in ecological and biogeochemical processes including carbon and nitrogen cycling, nutrient uptake, and also in soil formation (van der Heijden et al. 2008). The microbial community structure is affected by environmental factors such as pH, temperature, moisture, and nutrient content (Arcand et al. 2017; Lauber et al. 2009; Wang et al. 2014). In agricultural soils, these changes are dependent on the agricultural management practices, including tillage, crop rotation, fertilization, and pesticide application. Studies have established the effect of these practices on the microbial structure and soil metabolic capacity (Bronick and Lal 2005; Sun et al. 2016; Wang et al. 2014, 2017, 2019b). Bacterial community structure can also be altered according to the history of land use, land management practices, and edaphic properties (Jangid et al. 2011; Lauber et al. 2009).

Long-term chemical fertilizer use in conventional agriculture systems gives rise to substantial adverse effects on soil quality causing the reduction in the soil biological activities, loss of soil organic matter, and deterioration of the soil structure and nutrient recycling (Bronick and Lal 2005; Zhao et al. 2016). Besides, residues of pesticide can also cause dysbiosis in soil microbial biodiversity affecting the soil health irrespective of the region or soil types (Fang et al. 2018; Garcia-Delgado et al. 2019). Soil pH, geographical elevation, and mean annual temperature are some of the main factors that influence bacterial diversity (Lauber et al. 2009; Shen et al. 2019). Soil microbial profile significantly differs between organic and conventional farming systems, and organic farming supports a more stable and uniform community structure in comparison to conventional farming (Li et al. 2012; Wang et al. 2016).

The microbial extracellular enzymes in the soil play a prime role in ecosystem processes by catalyzing diverse reactions and also involved in carbon and nutrient cycling (Allison 2006). The dynamics and turn-over times of enzymes involved in enzyme-mediated decay vary with their function and origin depending on the abundance of the microbes (Schimel et al. 2017). Enzymatic activities are associated with soil properties including soil pH, bulk density, nutrient levels, and organic matter (Ai et al. 2012; Dai et al. 2018). Hydrolases, such as phosphatase, sulfatase, β-glucosidase, and urease, have been assessed as indices of P, S, C, and N cycling, respectively (Dai et al. 2018; Xian et al. 2015). These enzymes catalyze different substrates, releasing available inorganic forms of phosphates, sulfates, ammonia, and carbohydrates that serve as key energy sources for the plants and soil organisms. Due to their response to changing soil conditions, soil enzymes function as soil quality indicators and can be used to monitor soil health across agriculture management systems.

Tea (*Camellia sinensis* L. O. Kuntze) is one of the most economically valuable crops in the tropical and subtropical regions occupying a large share in the global market. Nitrogen fertilizers are extensively used in tea plantations to improve the yield and increase the amino acid content of the tea leaves (Watanabe 1995). This increased nitrogen fertilization leads to alteration of soil chemical composition, pH decrease, reduction of soil fungal diversity, and change of the fungal composition in tea plantations (Yang et al. 2019). Only a few studies have been conducted to understand the effect of different agriculture managements and fertilizer usage patterns on the microbial diversity in the tea plantations. A study comparing the soil bacterial community of tea plantations with the natural ecosystems such as wetland, grassland, and forest soils showed lower diversity in the tea soils (Lynn et al. 2017). Different mulching practices in tea plantations brought about variations in bacterial and fungal profiles showing organic mulching can improve the physicochemical properties of tea garden soils (Zhang et al. 2020).

However, the long-term effects of different fertilizer regimens and agricultural management on the microbial community structure and soil enzyme activity in tea plantation soil and ecosystem processes remains underexplored. Hence, in this study, the effects of long-term application of fertilizers and pesticides on the tea plantation soils under conventional management (20 years) and sustainable management (2 and 25 years) systems were compared. The study aimed to identify the indicators of soil quality by exploring the changes in soil enzyme activities, bacterial community structure and outline the factors that contribute to these changes.

## Materials and methods

### Field site and analysis of soil properties

The study was carried out in tea plantations (*C. sinensis* cv. Taicha 13) of the Mingjian Township (Nantou County, Taiwan) maintained under three different agricultural management practices namely conventional (CA; 23° 51′ N, 120° 38′ E; elevation 321 m; 0.126 ha), sustainable (SA; 23° 49′ N, 120° 39′ E; elevation 419 m; 0.194 ha), and transformational agriculture (TA; 23° 48′ N, 120° 39′ E; elevation 420 m; 0.488 ha) (Additional file 1: Fig. S1). The soils were all classified as Typic Hapludults according to the soil taxonomy of the United States Department of Agriculture. CA comprised chemical fertilizer application, including basal dressing one-time, top dressing four times, and pesticide application four times annually, and was maintained for 20 years. In the SA tea plantation, no herbicide, pesticide, or chemical fertilizer was applied for 25 years, but organic fertilizer was used instead. The organic fertilizer was applied in the field walk and plowed into the soil. In the TA tea plantation, conventional agriculture was followed for 28 years and then reformed into sustainable agriculture since 2015 (Additional file 2: Table S1). The fresh tea leaves were harvested from the CA, TA, and SA as 23,690, 1234, and 3455 kg/ha in 2016, and 20,212, 4381, and 1882 kg/ha in 2016 (Guo et al. 2017). Moreover, 45 species of plants including Asteraceae, Gramineae, Polygonaceae, Passifloraceae, Oxalidaceae, Commelinaceae, Amaranthaceae, Euphorbiaceae, Solanaceae, Dennstaedtiaceae, Araceae, Cyperaceae, Caryophyllaceae, Convolvulaceae, Lamiaceae, Fabaceae, Moraceae, Plantaginaceae, Scrophulariaceae, and Malvaceae were observed. The plant species in the CA, TA, and SA were 12, 42, and 43, respectively.

Soil samples were collected every two months between November 2016 and May 2017. The surface soils (depth 0–10 cm) from five plots of each treatment were sampled and pooled in sterile plastic tubes. The process was repeated three times. These samples were directly transported to the laboratory, sieved using a 2-mm mesh, and homogenized using a centrifugal ball mill (S100, Retsch, hahn, Germany) for analyzing enzymatic activity. The other samples were stored at − 20 °C until used for DNA extraction and chemical analysis. The pH and electrical conductivity (EC) of the soil (slurry 1:1, w/v) were measured. Organic matter was determined as a loss on the ignition after burning at 430 °C for 24 h. Total nitrogen was analyzed according to the Kjeldahl method. The extractable elements in soil including P, K, Ca, Mg, Fe, Mn, Cu, and Zn were determined using inductively coupled plasma (ICP) emission spectroscopy (Hendershot et al. 2008). Briefly, five-gram soil was added into Mehlich No.3 extracting solution and then shaken for 30 min. The mixture was filtered and performed by ICP analysis.

### Estimation of soil enzymatic activity

Acid phosphatase, arylsulfatase, and *β*-glucosidase activities were measured spectrophotometrically by determining *p*-nitrophenol released from *p*-nitrophenyl phosphate, *p*-nitrophenyl sulfate, and *p*-nitrophenyl-*β*-d-glucopyranoside, respectively (Eivazi and Tabatabai 1988; Ho 1979; Tabatabai and Bremner 1970). A mixture consisting of 0.5 g of soil, 0.2 mL of toluene, 0.5 mL of 115 mM *p*-nitrophenyl phosphate, and 2 mL of modified universal buffer (MUB) was used in the acid phosphatase activity assay and incubated at 37 °C for 1 h. For arylsulfatase assay, a mixture consisting of 0.5 g of soil, 0.2 mL of toluene, 0.5 mL of 25 mM *p*-nitrophenyl sulfate, and 2 mL of MUB was used and incubated at 37 °C for 1 h. For *β*-glucosidase activity, 0.2 mL of toluene, 0.5 mL of 25 mM *p*-nitrophenyl-*β*-d-glucopyranoside, and 2 mL of MUB were used and incubated at 37 °C for 1 h. MUB was composed of Tris, maleic acid, citric acid, boric acid, NaOH, HCl, and distilled water at pH 6.5 and was prepared according to the method of Tabatabai (Tabatabai 1982). The reaction for acid phosphatase and arylsulfatase activity was terminated by adding 2 mL of 0.5 M NaOH and 0.5 mL of 0.5 M CaCl_2_. The reaction for *β*-glucosidase activity was interrupted by adding 2 mL of 0.5 M Tris buffer (pH 12) and 0.5 mL of 0.5 M CaCl_2_. The mixture was filtered using filter paper (No. 393, Sartorius AG, Goettingen, Germany) and detected at OD_400_ by a spectrophotometer. For urease activity, 1 g of soil was mixed with 0.5 mL of 0.08 M urea and incubated at 37 °C for 2 h (Kandeler and Gerber 1988). The reaction was stopped by adding 10 mL of 1 N KCl. The filtrate of 200 μL was further mixed with 400 μL of 0.1% dichloroisocyanuric acid sodium salt, 1.8 mL of distilled water, and 1 mL of sodium salicylate (Na-salicylate/NaOH). The reaction mixture was incubated in the dark for 30 min and analyzed at OD_690_ by using a spectrophotometer. Different concentrations of *p*-nitrophenol and NH_4_Cl were used as standards for calculating enzymatic activities. The acetylene reduction method was used to determine soil N_2_-fixing activity (Hardy et al. 1973). An 80-mL tube filled with 17 mL of soil was incubated at 25 °C for 24 h under a gas mixture that had been substituted with 10% acetylene. Ethylene catalyzed by nitrogenase was measured in a 0.5-mL sample on a gas chromatography (GC) instrument (HITACHI model 163, Hitachi Ltd., Tokyo, Japan) equipped with a flame ionization detector (FID) and a packed column (1.0 m × 2.0 mm internal diameter, steel column packed with Porapak-T 80–100). The GC parameters were as follows: carrier gas, nitrogen; flow rate, 35 mL h^−1^; FID temperature, 110 °C; and column temperature, 65 °C.

### DNA sequencing of soil bacterial community by illumina MiSeq and bioinformatics analysis

To explore the soil microbial community, soil DNA sequencing was performed using next-generation sequencing. Soil DNA was extracted using the UltraClean Soil DNA Isolation Kit (MO BIO Laboratories, Inc., USA) and quantified as OD260/280 using a spectrophotometer. The hypervariable V6 region of the 16S rRNA gene was used for DNA amplification. PCR products were converted into blunt ends, and adapter ligation was performed according to the Beijing Genomics Institute (BGI) experimental workflow (Ju et al. 2017). Library construction, high-throughput sequencing, and genome de novo analysis were conducted by the BGI (BGI, Shenzen, China). The Agilent 2100 bioanalyzer instrument (Santa Clara, CA, USA) and real-time quantitative PCR were performed for quantification and qualification of the library. DNA sequencing was performed using a Miseq platform and the raw data were analyzed by the BGI bioinformatics workflow. The taxonomic ranks of microbes were assigned to operational taxonomic unit (OTU) representative sequence by using Ribosomal Database Project (RDP) at 97% similarity (Cole et al. 2014). The different samples combined with the OTU representative sequences were grouped using Venn diagrams (R software v3.1.1) (Foundation for Statistical Computing, Vienna, Austria). The principal component analysis (PCA) based on the OTU abundance performed using the ade4 package of R software. Visualization of the taxonomic distribution of microbes in a histogram was made using the R software. Heat maps for the species abundance were obtained using the gplots package of R software. Alpha diversity was calculated by Mothur (v1.31.2) including the observed species, Chao, ACE, Shannon, and Simpson indices.

### Statistical analysis

The data was presented as mean and standard deviation (SD). Duncan’s multiple range test at a confidence level of 95%, was determined with IBM SPSS Statistics v20 software package (SPSS Inc. Chicago, USA). Repeated measures analysis of variance (ANOVA) was performed to examine the interaction of temporal change and agricultural management affected by enzymatic activity. The relationship among the soil bacteria, enzymatic activities, and chemical properties was analyzed through redundancy analysis (RDA) performed using the vegan package of R software. ANOVA with multivariate linear regression was conducted to explore the differences in soil bacteria, enzymatic activities, and chemical properties among the sampled soils. Stepwise method (probability of F < 0.05) was used to interpret the responses of enzymatic activity and bacterial community to variance in soil properties.

### Nucleotide sequence accession numbers and data availability

This soil high-throughput DNA sequencing project was deposited at DDBJ/ENA/GenBank under accession numbers BioProject PRJNA545380 and BioSample SAMN11890669, SAMN11890668, SAMN11890645, SAMN11890644, SAMN11890643, SAMN11890627, SAMN11890626, SAMN11890625, SAMN11890494, SAMN11890481, SAMN11890470, and SAMN11890468.

## Results

### Soil properties along the tea plantations

**Fig. 1:**
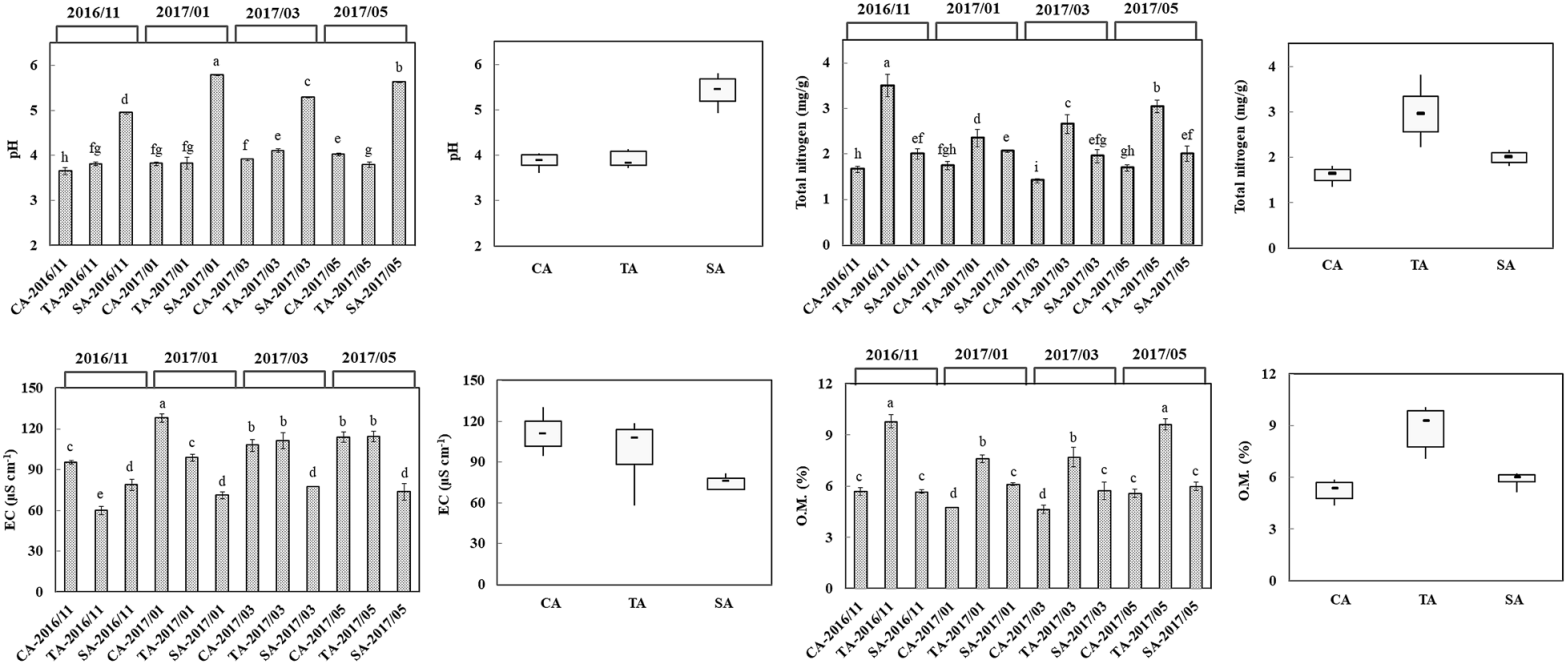
Soil pH, EC, total nitrogen, and organic matter of tea plantations. These parameters were determined from CA, TA, and SA soils between November 2016 and May 2017. Results are shown as mean ± standard deviation. The different letters labeled at columns are significantly different according to Duncan’s test (p < 0.05)

**Fig. 2:**
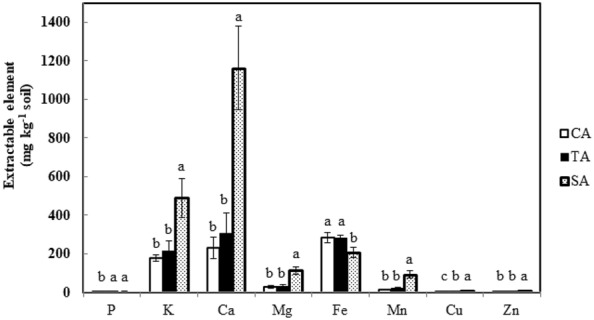
Soil extractable elements of tea plantations. The extractable elements including P, K, Ca, Mg, Mn, Cu, and Zn from the CA, TA, and SA soils were determined by ICP-MS. Results are shown as mean ± standard deviation. The different letters labeled at columns are significantly different according to Duncan’s test (p < 0.05)

**Fig. 3:**
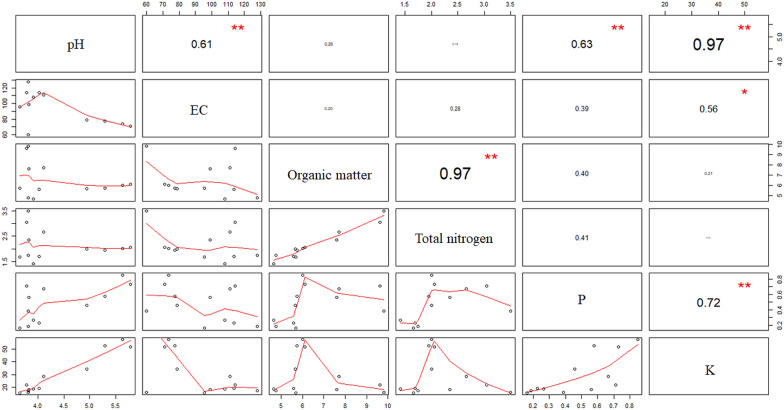
Pearson correlation among the studied soil parameters. The closer the value is to 1, the higher correlation between the two variables are. Significance is indicated by **p-value < 0.01, and *p-value < 0.05

The temperatures at the sampling times, November 8, 2016, January 5, 2017, March 15, 2017, and May 10, 2017, were 26.5 °C, 21.9 °C, 15.1 °C, and 29.7 °C, respectively. The soil properties are presented in Fig. 1. The SA soil had the highest overall pH (5.41) followed by TA and CA soils, while the trend was reversed in EC of the soil with SA having the lowest EC (75.2 μS cm^−1^). Total nitrogen and organic matter contents were the highest in TA soil followed by SA and CA soils. The extractable elements namely, P, K, Ca, Mg, Mn, Cu, and Zn in the SA soil were higher than that of the TA soil, followed by the CA soil (Fig. 2). According to the total nitrogen, organic matter, and extractable elements, the nutrients were not accumulated and fluctuated across the sampling time. On the other hand, the Pearson correlation coefficient was used to reflect the linear dependency between two random variables. Our result indicated that soil pH was positively correlated with soil P and K contents and negatively correlated with soil EC (Fig. 3). Also, a significant correlation was observed between organic matter and total N.

### Soil enzymatic activities among the tea plantations

**Fig. 4:**
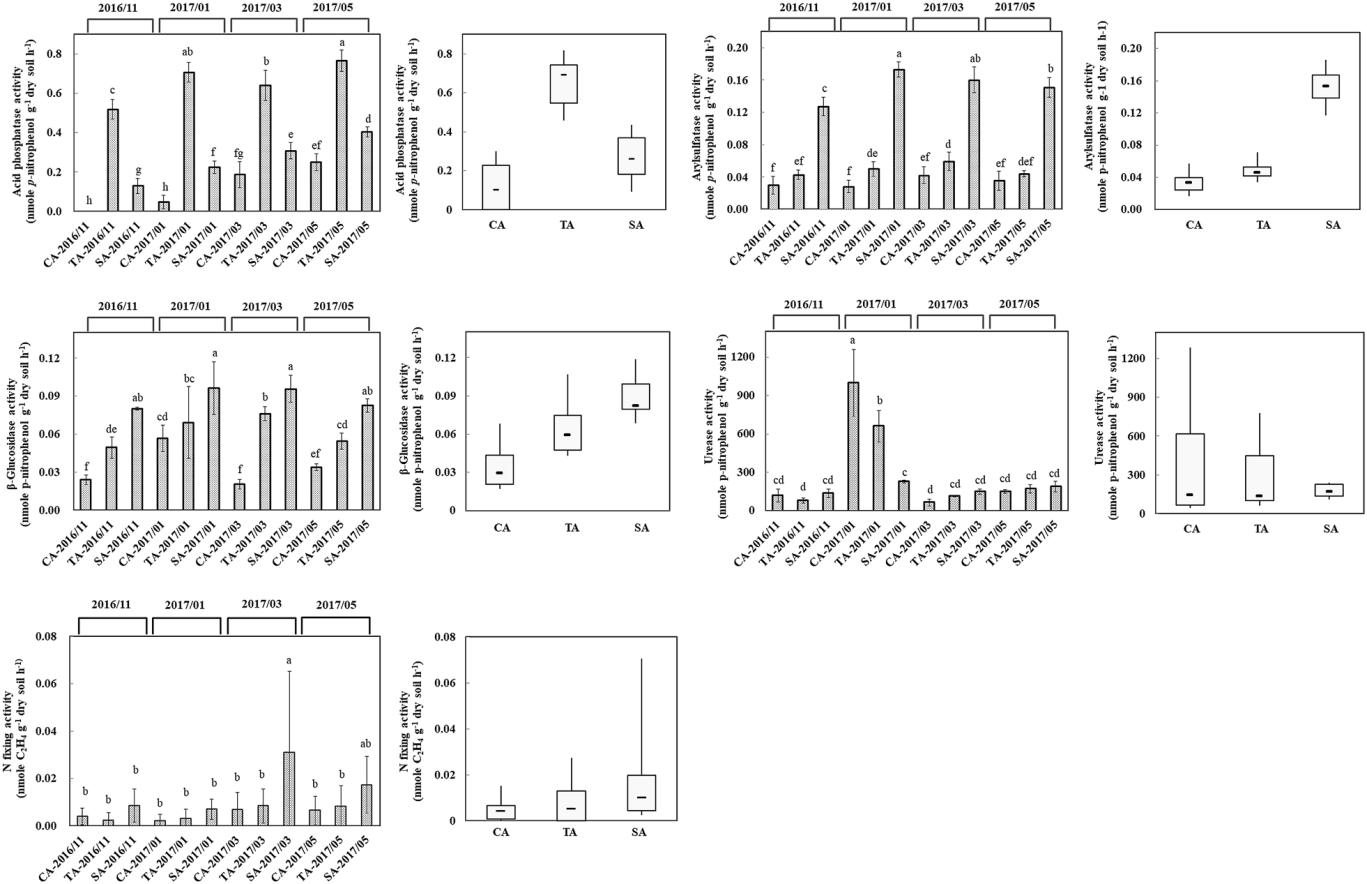
Soil enzymatic activities of tea plantations. Acid phosphatase, arylsulfatase, and *β*-glucosidase activities were determined according to the *p*-nitrophenol method. The nitrogen-fixing activity was determined by the acetylene reduction method using gas chromatography. Results are shown as mean ± standard deviation. The different letters labeled at columns are significantly different according to Duncan’s test (p < 0.05)

The activities of the enzymes, namely acid phosphatase, arylsulfatase, β-glucosidase, and urease, and nitrogen fixation involved in phosphorus, sulfur, carbon, and nitrogen cycles are presented in Fig. 4. Acid phosphatase in the TA soil was significantly higher than SA and CA soils (p < 0.001). Arylsulfatase and β-glucosidase activities were significantly higher in the SA soil (p < 0.001) than TA and CA soils. Though urease activity did not show a significant difference between the management practices (p = 0.003), the activity of CA soil was 4.3-times higher than that of SA soil in January 2017 sample. Nitrogen fixation was not significantly different across the agricultural practices; however, it was comparatively higher in the SA soil.

Repeated measures-ANOVA for the enzymatic activities was performed to evaluate the interaction between temporal changes and agricultural management practices (Table 1). Acid phosphatase, arylsulfatase, and urease activities showed a significant difference between agricultural management practices and time. The β-glucosidase activity showed marginally significant differences across sampling times and agricultural management practice. However, in the nitrogen-fixing activity, there were no significant changes in agricultural management systems along with the temporal scale.

**Table 1:** Statistical significance (p-value) for repeated measures ANOVA on acid phosphatase, arylsulfatase, β-glucosidase, urease, and nitrogen-fixing activity

	Enzymatic activity									
	Acid phosphatase		Arylsulfatase		β-Glucosidase		Urease		Nitrogen fixation	
	*F*	*p*	*F*	*p*	*F*	*p*	*F*	*p*	*F*	*p*
Test of within-subjects effects										
Time	53.709	0.000**	7.899	0.001**	8.476	0.009**	80.172	0.000**	2.283	0.174
Time × Management	3.246	0.016*	3.510	0.011*	2.918	0.081	18.060	0.001**	0.703	0.552
Test of between-subjects effects										
Intercept	6109.184	0.000**	4911.200	0.000**	1282.411	0.000**	243.964	0.000**	12.303	0.013*
Management	1297.194	0.000**	1116.072	0.000**	85.677	0.000**	7.665	0.022*	2.102	0.203

Significance is indicated by **p-value < 0.01, and *p-value < 0.05

### Changes in the soil bacterial community structure

**Fig. 5:**
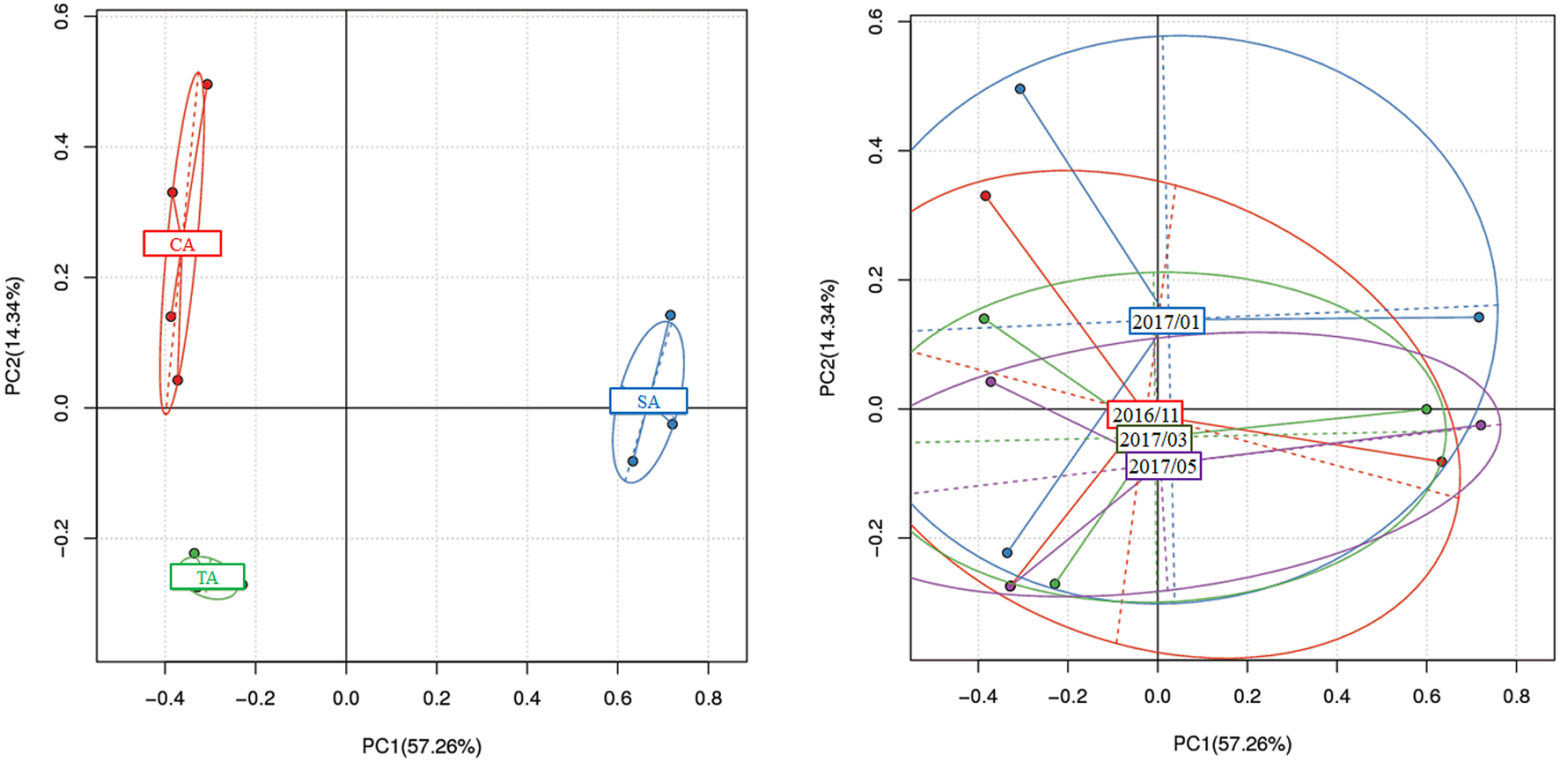
Principal component analysis of OTUs obtained from CA, TA, and SA soils between November 2016 and May 2017. The x-axis and y-axis indicate principal component 1 (PC1) and principal component 2 (PC2), respectively. The clustering groups of samples are based on different agricultural management and sampling times. The PCAs were implemented by the R software

To estimate the diversity of bacterial distribution in soil, the total eubacterial community was examined using high-throughput sequencing. After raw reads were trimmed and chimeric reads were removed, the average clean reads and clean data of each sample were 108,342 reads and 31.125 Mb, respectively (Additional file 3: Table S2). The OTUs obtained for the different agricultural management practices and sampling times are represented as a Venn diagram (Additional file 4: Fig. S2). The number of unique OTUs in the SA soil was significantly higher than those of the CA and TA soils. Based on the sampling time, the order of presence of unique OTUs is May 2017 > November 2016 > March 2017 > January 2017. PCA revealed that the TA soil clustered closely with the CA soil but was distinct from the SA soil (57.26%) (Fig. 5). However, no grouping was observed for the sampling times.

**Fig. 6:**
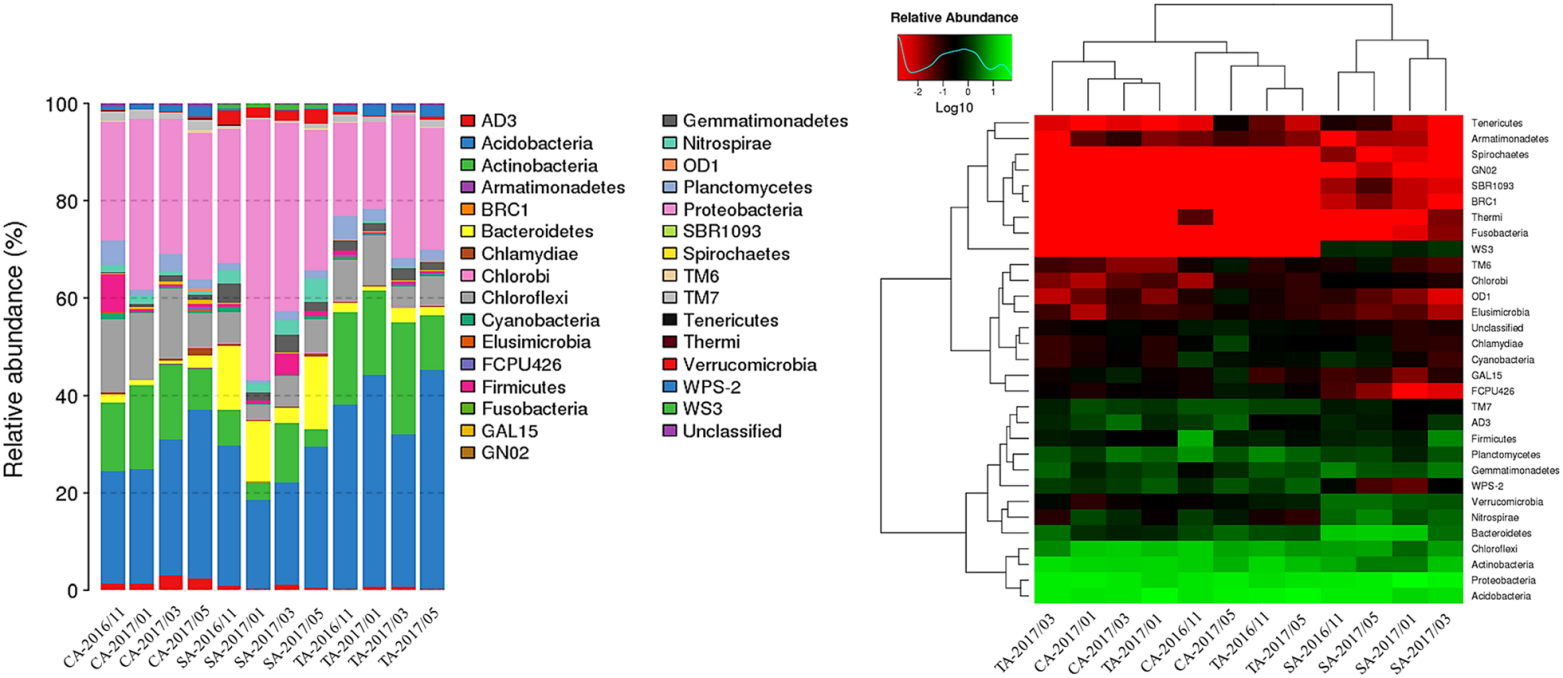
Distribution and heat map of OTUs at the phylum level obtained from CA, TA, and SA soils between November 2016 and May 2017. The taxonomic composition distribution and heat map were implemented by the R software

The soil community structure at the phylum level showed that Proteobacteria, Acidobacteria, Actinobacteria, Chloroflexi, and Bacteroidetes were the predominant phyla (Fig. 6). The relative abundance of Bacteroidetes in the SA soil was 7.3 and 6.0-fold higher than that in the CA and TA soils, respectively and the differences are statistically significant (p < 0.01; p < 0.05). Gemmatimonadetes and Nitrospirae were also higher in SA soil compared to soils with other management practices. By contrast, the relative abundance of Actinobacteria in the SA soil was significantly lower than the TA and CA soils (p < 0.01; p < 0.001). The abundance of Chloroflexi was significantly lower in SA soil compared to CA soil (p < 0.05). The distribution of the major phyla among the management practices and sampling times are given as supplementary figure (Additional file 5: Fig. S3). Further, at the class level, Gammaproteobacteria was dominant having a relative abundance of 17.3% and 15.1% in the CA and SA soils respectively. In the TA soil, Alphaproteobacteria was dominant with a relative abundance of 13.2%. The heat map showing the clustering based on the abundance of each phylum is shown in Fig. 6. Three clades are clearly divided and the SA soil is clustered in the same group. The CA and TA soils sampled in November 2016 and May 2017, and January 2017 and March 2017 showed the distribution of the bacterial community in two distinct clades.

**Fig. 7:**
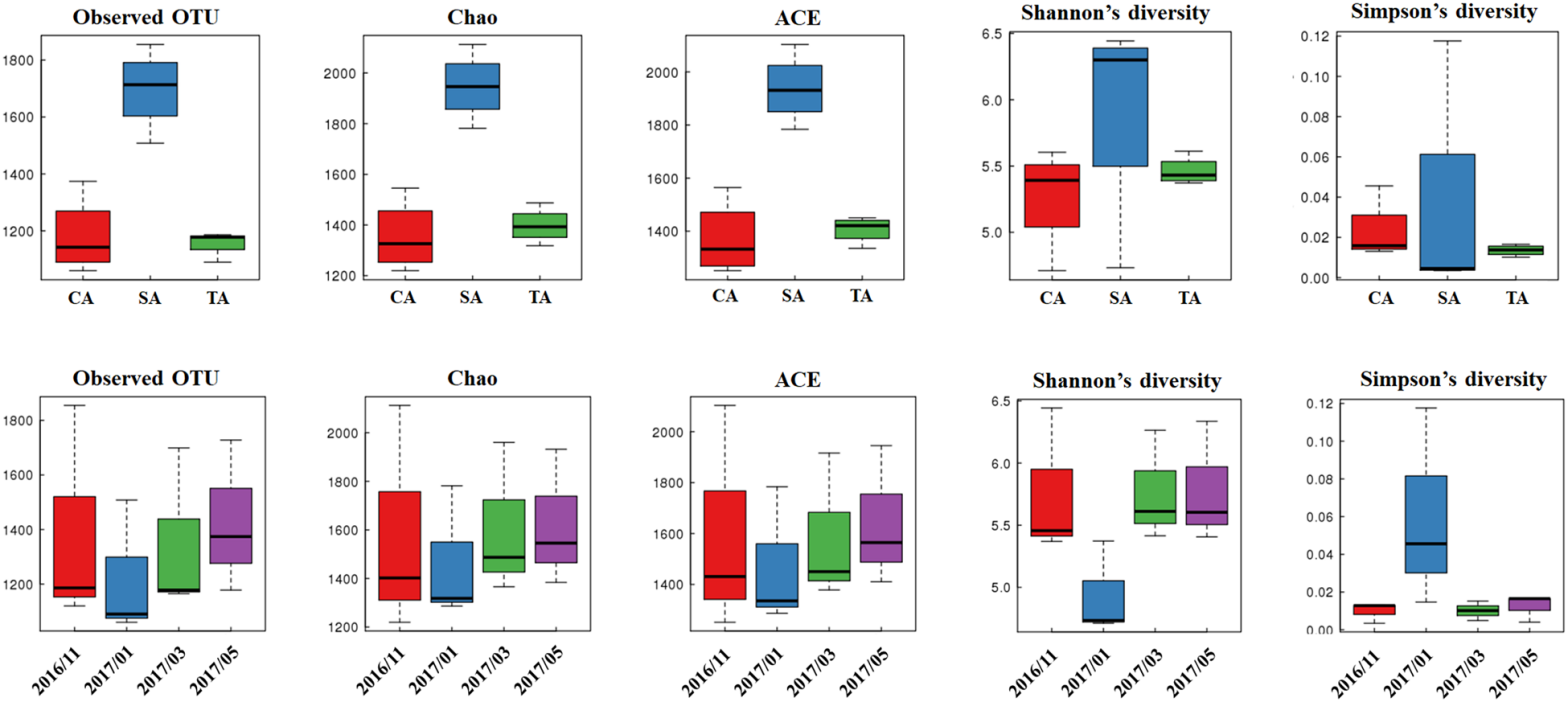
Alpha diversity of the soil bacteria in the tea plantations. The alpha diversity including observed OTU, Chao, ACE, Shannon, and Simpson from CA, TA, and SA soils between November 2016 and May 2017 are given. The indices are calculated by Mothur (v1.31.2) and implemented by the R software

Bacterial species abundance and diversity estimates such as observed specie richness, SOBS, Chao, ACE, Shannon, and Simpson diversity indices across the management and sampling times are shown in Fig. 7. The SA soil had a higher number of bacterial species and greater community richness than the soils managed under TA and CA practices. This finding is consistent with the species diversity displaying high Shannon and low Simpson indices in the SA soil. The bacterial species and community richness did not differ considerably with the sampling time.

### Correlation between bacterial community, soil enzymatic activity, and soil properties

**Fig. 8:**
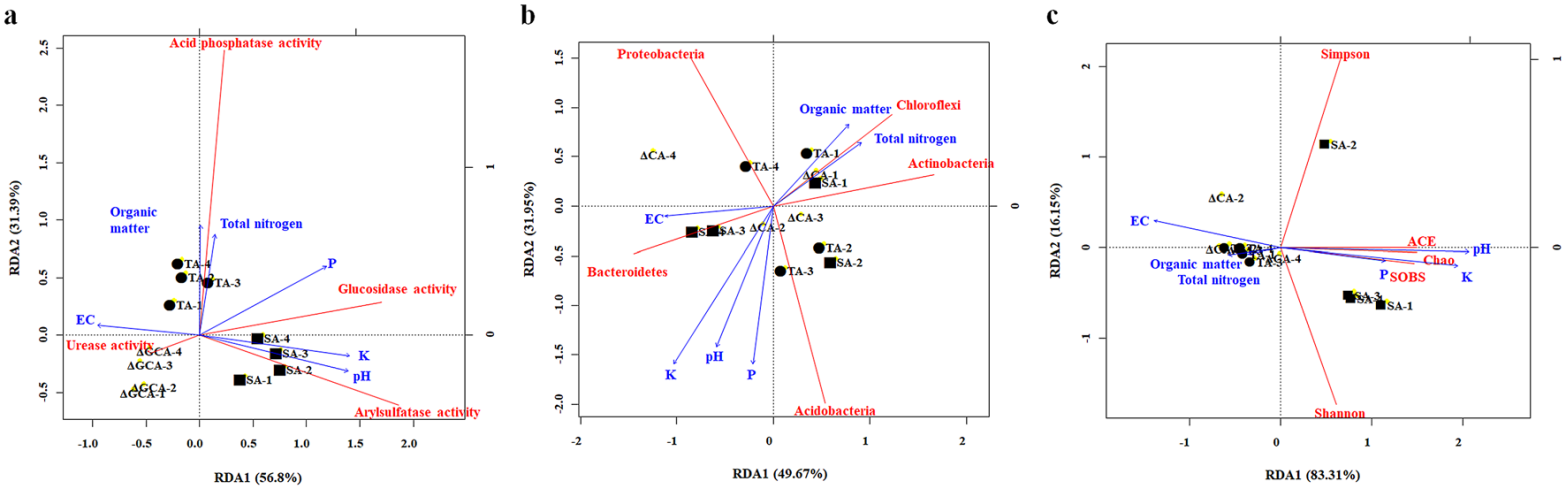
Redundancy analysis for the soil parameters of the studied tea plantations. The correlation between the soil properties, and (**a**) enzymatic activities, (**b**) the most abundant bacterial phylum, and (**c**) alpha diversity of species was respectively analyzed by RDA. The RDAs were implemented by the R software

Redundancy analysis was used to explore the relationship between bacterial community, soil enzymatic activities, and soil properties. The RDA components explain 88.19%, 81.62%, and 99.46% of the variation in enzymatic activity, bacterial composition, and bacterial alpha diversity, respectively from the data obtained for the soil samples (Fig. 8). For the enzymatic activity, the first component explained 56.8% of the total variation and also, separated the CA, TA, and SA samples. Arylsulfatase activity was dominant in the SA soil and was related to the pH changes, while the acid phosphatase activity was predominant in the TA soil and is associated with the total nitrogen and organic matter of the soil. On the other hand, urease activity was dominant in the CA soil and was related to the EC value. Further, for the bacterial community, an association was seen between available phosphorus, soil pH, and the abundance of Acidobacteria. Additionally, Chloroflexi and Actinobacteria were positively correlated with total nitrogen and organic matter; however, they were negatively associated with the soil EC. According to their close groupings, Acidobacteria and Proteobacteria occurred predominantly in the TA soil, while Chloroflexi and Actinobacteria were predominant in all the soils in November 2016. The observed species richness of bacterial community clustered along RDA axis-2, which is highly correlated with soil pH, K, and P and negatively correlated to soil EC.

Pearson correlation between enzymatic activity, bacterial community, and soil properties is shown in Table 2. A significant difference was observed between different enzymatic activity and soil properties. Moreover, the observed species and abundance indices including SOBS, Chao, and ACE were significantly related to soil pH. Nevertheless, no significant relationship was perceived in the dominant bacteria and bacterial diversity such as Acidobacteria, Actinobacteria, Bacteroidetes, Proteobacteria, Shannon, and Simpson index. Chloroflexi showed a significant negative correlation (p-value < 0.05) with EC. Multivariate linear regression ANOVA with the stepwise method was performed for modeling the enzymatic activity and bacterial community with soil chemical properties. To identify the major factors that influence the enzymatic activity, observed species, and bacterial abundance, the soil properties were used as independent variables for the stepwise multiple regressions. Based on this the regressions are constructed (Additional file 6: Table S3 (Eqs. 1–6)).

**Table 2:** Pearson correlation between enzymatic activity, bacterial community, and soil properties

Pearson correlation	pH	EC	Organic matter	Total nitrogen	p	K
Acid phosphatase	– 0.157	0.004	0.822**	0.757**	0.619*	– 0.037
Arylsulfatase	0.981**	– 0.663**	– 0.209	– 0.124	0.655*	0.962**
β-Glucosidase	0.784**	– 0.463	0.077	0.190	0.761**	0.804**
Urease	– 0.207	0.447	– 0.199	– 0.156	– 0.193	– 0.222
Acidobacteria	0.293	0.019	– 0.124	– 0.044	0.376	0.290
Actinobacteria	– 0.254	– 0.148	0.159	0.130	– 0.141	– 0.348
Bacteroidetes	0.414	– 0.089	– 0.282	– 0.298	0.202	0.508
Chloroflexi	0.020	– 0.559*	0.190	0.238	– 0.128	– 0.079
Proteobacteria	– 0.325	0.308	0.115	0.076	– 0.323	– 0.326
SOBS	0.852**	– 0.591*	– 0.279	– 0.196	0.434	0.808**
Chao	0.872**	– 0.577*	– 0.235	– 0.132	0.505*	0.832**
ACE	0.871**	– 0.599*	– 0.223	– 0.127	0.501*	0.823**
Shannon	0.381	– 0.396	– 0.042	– 0.019	0.295	0.438
Simpson	0.361	– 0.094	– 0.144	– 0.106	0.141	0.246

Significance is indicated by **p-value < 0.01, and *p-value < 0.05

## Discussion

Different agricultural management practices used in the tea plantation have a greater impact on the soil quality particularly on the biological parameters. The soil enzymes and bacterial composition are assumed to be indicators of soil quality (Acosta-Martinez et al. 2018; Arafat et al. 2017; Jeanbille et al. 2016), and the results of this study provide valuable insights into the relationship among soil physic-chemical properties, enzymatic activity, and bacterial community derived from the field data that included soil samples from three different agricultural management practices over the temporal scale. In the present study, the SA soil was treated without herbicides or pesticides and chemical fertilizer application; consequently, this soil exhibited a higher pH, extractable elements, arylsulfatase, β-glucosidase, nitrogen fixation, bacterial richness, and bacterial diversity than the other soils. This corresponded to a low EC value in the SA soil. Because of the long-term application of chemical fertilizers, acidification reduced the pH of the CA and TA soils even though the TA soil had been reformed into a sustainable system since 2015. This shows that soil health recovery takes a longer time after shifting to more sustainable practices. One of the key reasons may be time taken for the restoration of the healthy microbial community structure and the microbial processes. However, the recovery of soil microbial community on transitioning from chemical farming to organic farming remains to be studied. This provides evidence for the sustainability of this practice in maintaining soil health. Long-term tea cultivation with the application of chemical fertilizers is known to significantly reduce the soil pH and bacterial composition (Arafat et al. 2017; Li et al. 2016).

Several enzymatic activities are involved in the soil process and are considered critical parameters of soil health (Dai et al. 2018). Acid phosphatase and arylsulfatase can catalyze the hydrolysis of various substrates attached to free phosphoryl and sulfate groups (Pettit et al. 1977; Tang et al. 2013). The enzyme β-glucosidase catalyzes the glycosidic bonds from complex carbohydrate molecules to terminal non-reducing residues, and urease hydrolyzes urea to form ammonia and carbon dioxide through ureolysis (Chae et al. 2017; Oshiki et al. 2018). Nitrogen fixation is accomplished by bacteria and archaea encoding nitrogenase complexes for ammonification (Stein and Klotz 2016). These processes are required to maintain a balance in the nutrient cycling and supply of available nutrients to the plantation. Soil enzymatic activity was greater in the SA system compared to CA for all enzymes except acid phosphatase that showed higher activity in the CA soil. Acid phosphatase activity is favored by the acidic condition. Repeated measures ANOVA carried out within and between treatment across time, reflected the difference of enzymatic activity between three agricultural managements. The result demonstrated that the activity of acid phosphatase, arylsulfatase, and urease was significantly affected by agricultural management practices across temporal scale, while the difference in the nitrogen fixation rate was not statistically significant among the practices. The β-glucosidase activity was sensitive to time and management practice but no clear response was repeated in the interaction of time and treatment.

According to the RDA analysis, the distinction between enzymatic activities, soil properties, and agricultural management practices was extremely clear. A close association of acid phosphatase activity with organic matter and total nitrogen was observed. This result was similar to the findings in the hardwood forests soil and the surface sediments of *Ctenopharyngodon idellus* aquaculture ponds, showing the changes in C/N ratio in response to acid phosphatase activity (Dai et al. 2018). β-Glucosidase is known to be associated with the carbon cycle and also influenced by the level of organic carbon in the soil (Dai et al. 2018; Xian et al. 2015). However, the correlation coefficient between β-glucosidase activity and the extractable elements such as K and P were higher than that of organic matter in this study. This was also reflected in the arylsulfatase activity that was correlated to the soil pH and K in the SA soil.

To identify the influence of soil properties on each enzymatic activity, multiple regression with the stepwise method was utilized. Under the probability of F < 0.05, acid phosphatase, arylsulfatase, and β-glucosidase were considered for the significance level of variables. Among the six quantified soil properties, the multiple regression model picked out organic matter, pH, and K content as significant predictors of acid phosphatase, arylsulfatase, and β-glucosidase activities respectively (Additional file 6: Table S3 (Eqs. 1–3)). The soil pH as a significant predictor explained 96.3% of the variation in arylsulfatase activity, indicating that high soil pH might lead to high arylsulfatase activity or vice versa. On the other hand, the organic matter and potassium content explained 67.6% and 64.6% of the variation in acid phosphatase and β-glucosidase activities, respectively. Combining the RDA and multiple regression analysis, the arylsulfatase activity could be suggested as the soil quality index for sustainable management. This result was corresponding to the arylsulfatase activity of heavy metal contaminated soil as the indicator (Xian et al. 2015). The arylsulfatase was not only sensitive to the adverse impacts of Pb-, As-, and Cd-polluted soils, but also the low pH soils. Therefore, the arylsulfatase activity was attractive as the indicator for monitoring the acidified soil.

To compare the composition and distribution of bacterial communities among the soils under CA, TA, and SA systems, a culture-independent technique of high-throughput sequencing was applied. This technique provides a large amount of information at the molecular level and is directly used to interpret the microbial diversity (Arafat et al. 2017; Lauber et al. 2009). This study showed that Proteobacteria, Acidobacteria, and Actinobacteria to be the most abundant eubacteria, corresponding to previous characterizations of the bacterial consortia in the tea plantations (Arafat et al. 2017; Wang et al. 2019a). Proteobacteria, Acidobacteria, and Actinobacteria constituted respectively 29.3%, 27.4%, and 13.8% in the CA soil; 22.8%, 39.7%, 17.5% in the TA soils; and 37.1%, 24.3%, and 6.8% in the SA soil.

Proteobacteria had a higher relative abundance in SA soil followed by TA and CA which is in concordance with another microbial characterization of tea plantation. Proteobacteria are the highly diverse group of bacterial phyla playing a major role in the biogeochemical cycle (Spain et al. 2009) and its abundance in the soil managed using SA practices indicate a higher availability of available nutrients in this soil. However, Bacteroidetes constituted a comparatively higher proportion in the SA soil (10.9%) than in the CA (1.5%) and TA (1.8%) soils. Higher abundance of Bacteroidetes was closely associated with increased soil moisture content in a 30-year-old tea plantation (Arafat et al. 2017) while showing a positive correlation with soil pH and available K (Zhao et al. 2014). It was also significantly higher in organically cultivated lands compared to chemically cultivated land (Chaudhry et al. 2012). Bacteroidetes also showed a positive correlation with pH, K, and P and a negative correlation with EC content of the soil. Thus, high Bacteroidetes content in SA soil can be due to its differences in the soil properties along with the lack of application of chemical fertilizer. Contrasting reports exist on the relative abundance of Acidobacteria that is negatively associated with the pH in the soil of different aged tea plantations (Wang et al. 2019a). However, Arafat et al. observed a positive correlation between root-associated Acidobacteria and soil pH in the tea plantation (Arafat et al. 2017; Zhou et al. 2015). The changes in Acidobacteria abundance may be due to the influence of other edaphic factors. In this study, the distribution of Acidobacteria was not only positively correlated with soil pH but also with P content. Nitrospirae, the bacterial phylum containing ammonia-oxidizing and nitrate-oxidizing bacteria was significantly higher in SA soil and lower abundance in other soils can be due to the addition of N fertilizers as indicated in other studies (Wang et al. 2018).

The bacterial species diversity is also an important parameter changing with the soil biotic and abiotic conditions. As evident, the alpha diversity analysis showed higher bacterial species richness and diversity in the SA soils. The positive relationships among bacterial species richness, soil pH, K, and P contents in the SA soils are also interesting observations from this study. Phosphorous is the primary prerequisite for an organism’s growth because it is involved in energy transfer, biological oxidation, cell division, and other cellular metabolic activities. Inorganic phosphates are complex with iron and aluminum in acidic soils such as the soils in tea plantations, and P becomes limited for organisms; furthermore, its content is a crucial factor affecting bacterial abundance (Chen et al. 2006). Moreover, acidic soils can result in the loss of extractable elements, such as K, Ca, and Mg that are required for the plant (Rafael et al. 2018). The SA soil with high pH was higher in the content of extractable elements than in the CA and TA soils, except for Fe. This is also confirmed by the Pearson correlation indicating that the bacterial richness indices such as SOBS, Chao, and ACE index had a significant correlation with pH, EC, P, and K contents. However, almost no significant difference between the dominant phyla, Shannon and Simpson index, and soil properties was observed.

Under the probability of F < 0.05, SOBS, Chao, and ACE indices were considered for the significance level of variables. Among the six quantified soil properties, the multiple regression model only picked out pH as a significant predictor of SOBS, Chao, and ACE (Additional file 6: Table S3 (Eqs. 4–6)). The soil pH as a significant predictor explained over 72.5% of the variation in the bacterial richness, indicated that high soil pH towards the neutral range might favor higher bacterial abundance. Correlation and regression analysis between soil properties and microbial communities collectively revealed pH as an indicator of soil quality that positively influences the bacterial richness and enzymatic activity but the dominant phyla were not suitable as soil quality index for sustainable management.

From the cluster heat map analysis using the bacterial abundance data at the phylum level across the soil management practices and time scale, three distinct clades were observed representing the distribution of bacterial community according to the soil. The soil samples from SA collected at different sampling times revealed a highly similar bacterial composition. However, the CA and TA soils were divided into two clusters sampled in January 2017 and March 2017, and in November 2016 and May 2017. Although the time-related variation in the bacterial composition of the CA and TA soils was in agreement with the findings of Calbrix et al. (2007), it was not reflected in SA, suggesting that soil pH had a greater effect on bacterial composition than time variation. Variation in the temperature and other seasonal changes are also considered to be significant factors in microbial community composition (Hayden et al. 2012). Moreover, several studies have indicated that sampling time significantly affected bacterial abundance, which was low in the summer because of a seasonal effect (Jung et al. 2012; Rasche et al. 2011). In the present study, the Shannon and Simpson diversity indices were comparatively lower in January 2017 than the other sampling times and contrary to the results of the previous studies (Jung et al. 2012; Rasche et al. 2011). The low bacterial abundance and diversity were observed in the winter with gradually decreased temperature conditions.

## Conclusions

The study provided evidence for the influence of different agricultural management practices on soil enzymes and microbial diversity in tea plantations. Enzymes such as acid phosphatase, arylsulfatase, and urease are sensitive and get influenced by the agricultural management practices and temporal changes in the tea plantation. Further, the acid phosphatase, arylsulfatase, and β-glucosidase enzyme activities are affected by the soil organic matter, pH, and K respectively. Soil pH was shown to be a significant predictor of species richness (SOBS, Chao, and ACE) and was also associated with arylsulfatase activity which can be suitable as the soil quality index for monitoring sustainable management. The soils under organic management practice (SA) can harbor higher bacterial species richness and diversity indicative of enhanced ecosystem functioning and nutrient availability. On contrary, a decline in soil pH resulting in reduced bacterial abundance and enzymatic activities because of the long-term application of chemical fertilizer was seen in CA soil. The effect of agricultural management on bacterial composition was greater than the temporal variations in the bacterial composition, diversity, and enzymatic activities. Based on the cross-over analysis of the bacterial composition, enzymatic activity, and other soil properties, the relationship between bacterial composition and biologically-driven ecological processes can be defined for the tea plantation. Extensive multi-centric research studies should be undertaken by exercising uniform protocols to establish the most suitable farming practice that can improve the crop yield and maintain resilient soil health of tea plantations.

## Supplementary Information

**Additional file 1: Fig. S1**. Geographical maps of three tea plantations including CA, TA, and SA managements. The red frames indicate the experimental fields. The maps were obtained from the Google map.

**Additional file 2: Table S1**. The agricultural management practices from conventional (CA), transformational (TA), and sustainable (SA) agriculture between 2016–2017.

**Additional file 3: Table S2**. Summary of 16S rRNA reads from soil DNA extracted from CA, TA, and SA soils between November 2016 and May 2017 according to the Illumina MiSeq analysis.

**Additional file 4: Fig. S2**. Venn diagram representing the OTUs obtained from CA, TA, and SA soils between November 2016 and May 2017. The diagrams were implemented by the R software.

**Additional file 5: Fig. S3**. Box plot showing (a) the distribution top six phyla at three soils and (b) at different sampling times.

**Additional file 6: Table S3**. Modeling the enzymatic activity and bacterial community with soil chemical properties by multivariate linear regression ANOVA with the stepwise method.

## Data Availability

The data used and analyzed in this study can be provided from the corresponding author for scientific, non-profit purpose.
