# Supplementary material for: Agricultural management practices influence the soil enzyme activity and bacterial community structure in tea plantations
Source: Bot Stud. 2021 May 18;62:8. doi: 10.1186/s40529-021-00314-9 (PMC8131499; doi:10.1186/s40529-021-00314-9)
Supplement: Supplementary file 1 — Additional file 1: Fig. S1. Geographical maps of three tea plantations including CA, TA, and SA managements. The red frames indicate the experimental fields. The maps were obtained from the Google map. [file 40529_2021_314_MOESM1_ESM.docx]

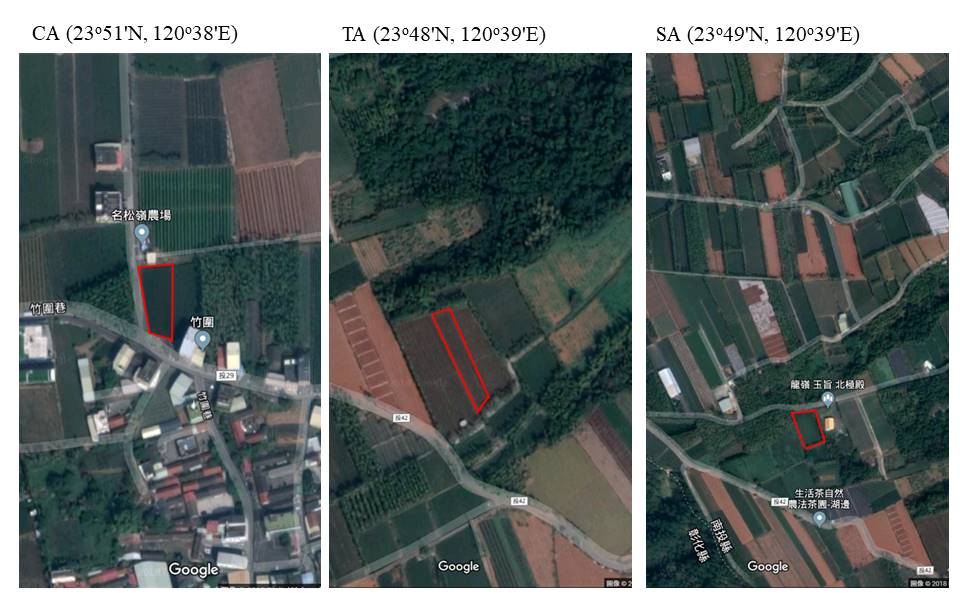


Fig. S1. Geographical maps of three tea plantations including CA, TA, and SA managements. The red frames indicate the experimental fields. The maps were obtained from the Google map.
