# Supplementary material for: Agricultural management practices influence the soil enzyme activity and bacterial community structure in tea plantations
Source: Bot Stud. 2021 May 18;62:8. doi: 10.1186/s40529-021-00314-9 (PMC8131499; doi:10.1186/s40529-021-00314-9)
Supplement: Supplementary file 2 — Additional file 2: Table S1. The agricultural management practices from conventional (CA), transformational (TA), and sustainable (SA) agriculture between 2016–2017. [file 40529_2021_314_MOESM2_ESM.docx]

Table S1. The agricultural management practices from conventional (CA), transformational (TA), and sustainable (SA) agriculture between 2016-2017.

| Management practices | CA | TA | SA |
| --- | --- | --- | --- |
| Chemical fertilizer | Taifer 142 fertilizer (N:P_2_O_5_:K_2_O:MgO = 23:6:10:3), 80-100 kg/one-time | none | none |
| Application of chemical fertilizer | Dressing one-time and top dressing four times | none | none |
| Pesticide | Methomyl, Difenoconazole, Spinetoram, Abamectin | none | none |
| Organic fertilizer | N:P_2_O_5_:K_2_O:organic matter = 3:3:2.4:78. Solid form. Basal-dressing fertilizer one-time. 1000 kg/0.1 ha. | N:P_2_O_5_:K_2_O:organic matter = 5:2.5:2.5:80. Solid form. Basal-dressing fertilizer one-time. 1000 kg/0.1 ha. | N:P_2_O_5_:K_2_O:organic matter = 5:2.5:2.5:80. Solid form. Basal-dressing fertilizer one-time. 1000 kg/0.1 ha. |
