# Supplementary material for: Agricultural management practices influence the soil enzyme activity and bacterial community structure in tea plantations
Source: Bot Stud. 2021 May 18;62:8. doi: 10.1186/s40529-021-00314-9 (PMC8131499; doi:10.1186/s40529-021-00314-9)
Supplement: Supplementary file 3 — Additional file 3: Table S2. Summary of 16S rRNA reads from soil DNA extracted from CA, TA, and SA soils between November 2016 and May 2017 according to the Illumina MiSeq analysis. [file 40529_2021_314_MOESM3_ESM.docx]

Table S2. Summary of 16S rRNA reads from soil DNA extracted from CA, TA, and SA soils between November 2016 and May 2017 according to the Illumina MiSeq analysis.

| Samples | Reads length (bp) | Average length (bp) | Raw data (Mb) | Clean data (Mb) | Raw reads | Clean reads | OUT number |
| --- | --- | --- | --- | --- | --- | --- | --- |
| CA-2016/11 | 296/300 | 415 | 35.42 | 30.93 | 59435×2 | 53860×2 | 1120 |
| TA-2016/11 | 297/299 | 412 | 36.13 | 31.40 | 60618×2 | 54855×2 | 1186 |
| SA-2016/11 | 289/299 | 417 | 35.16 | 31.37 | 58887×2 | 54432×2 | 1855 |
| CA-2017/01 | 299/299 | 416 | 35.63 | 31.18 | 59578×2 | 54287×2 | 1060 |
| TA-2017/01 | 300/299 | 412 | 36.17 | 31.16 | 60382×2 | 54193×2 | 1090 |
| SA-2017/01 | 300/300 | 421 | 34.92 | 31.44 | 58203×2 | 54315×2 | 1508 |
| CA-2017/03 | 293/300 | 413 | 36.54 | 30.86 | 61616×2 | 54212×2 | 1165 |
| TA-2017/03 | 294/300 | 412 | 34.86 | 30.82 | 58690×2 | 53824×2 | 1178 |
| SA-2017/03 | 297/300 | 417 | 34.75 | 31.09 | 58208×2 | 54009×2 | 1699 |
| CA-2017/05 | 297/300 | 414 | 35.94 | 31.31 | 60204×2 | 54514×2 | 1374 |
| TA-2017/05 | 298/300 | 412 | 34.97 | 30.81 | 58475×2 | 53369×2 | 1178 |
| SA-2017/05 | 299/300 | 418 | 36.85 | 31.13 | 61519×2 | 54183×2 | 1728 |
