# Supplementary material for: Agricultural management practices influence the soil enzyme activity and bacterial community structure in tea plantations
Source: Bot Stud. 2021 May 18;62:8. doi: 10.1186/s40529-021-00314-9 (PMC8131499; doi:10.1186/s40529-021-00314-9)
Supplement: Supplementary file 4 — Additional file 4: Fig. S2. Venn diagram representing the OTUs obtained from CA, TA, and SA soils between November 2016 and May 2017. The diagrams were implemented by the R software. [file 40529_2021_314_MOESM4_ESM.docx]

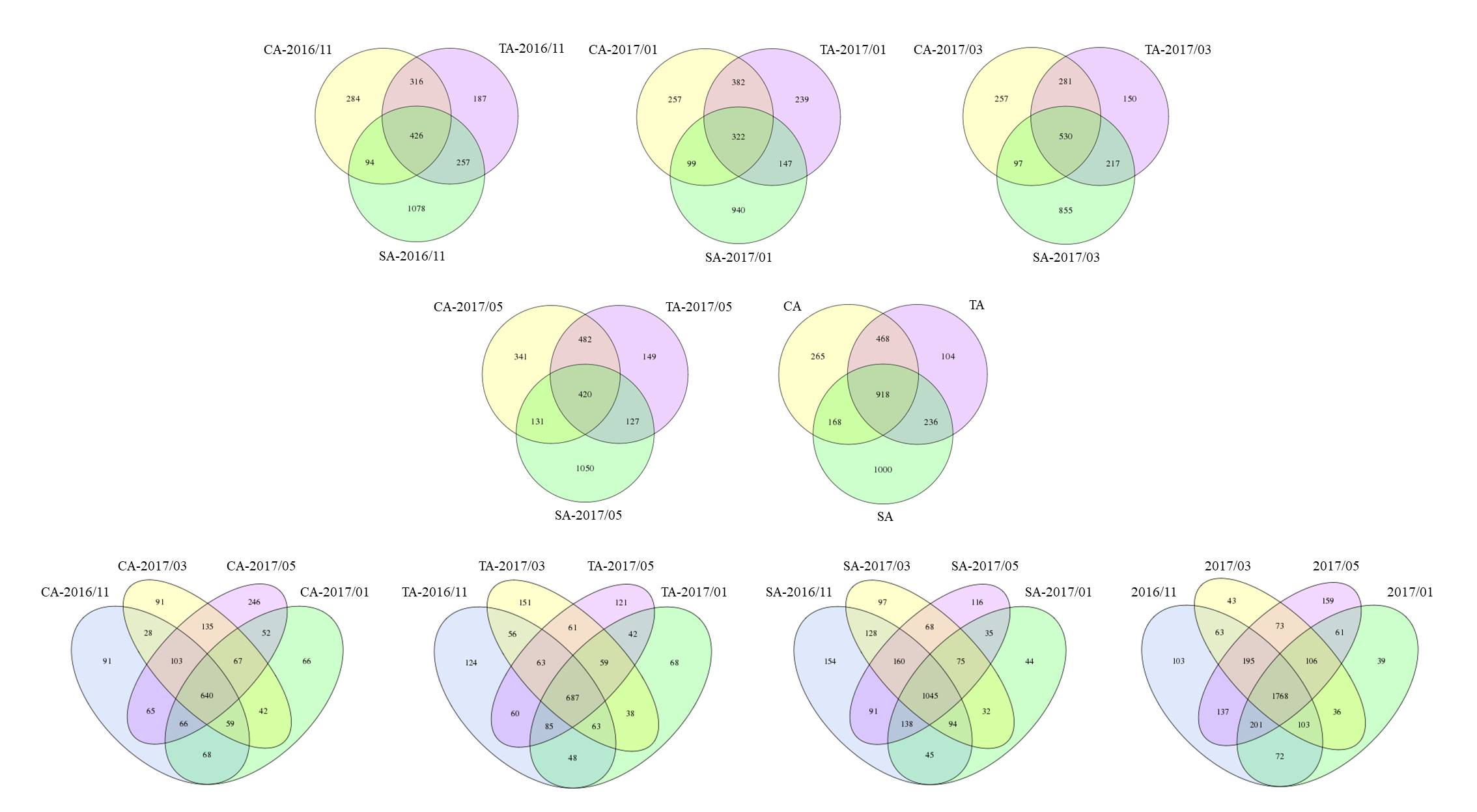


Fig. S2. Venn diagram representing the OTUs obtained from CA, TA, and SA soils between November 2016 and May 2017. The diagrams were implemented by the R software.
