# Supplementary material for: Agricultural management practices influence the soil enzyme activity and bacterial community structure in tea plantations
Source: Bot Stud. 2021 May 18;62:8. doi: 10.1186/s40529-021-00314-9 (PMC8131499; doi:10.1186/s40529-021-00314-9)
Supplement: Supplementary file 5 — Additional file 5: Fig. S3. Box plot showing (a) the distribution top six phyla at three soils and (b) at different sampling times. [file 40529_2021_314_MOESM5_ESM.docx]

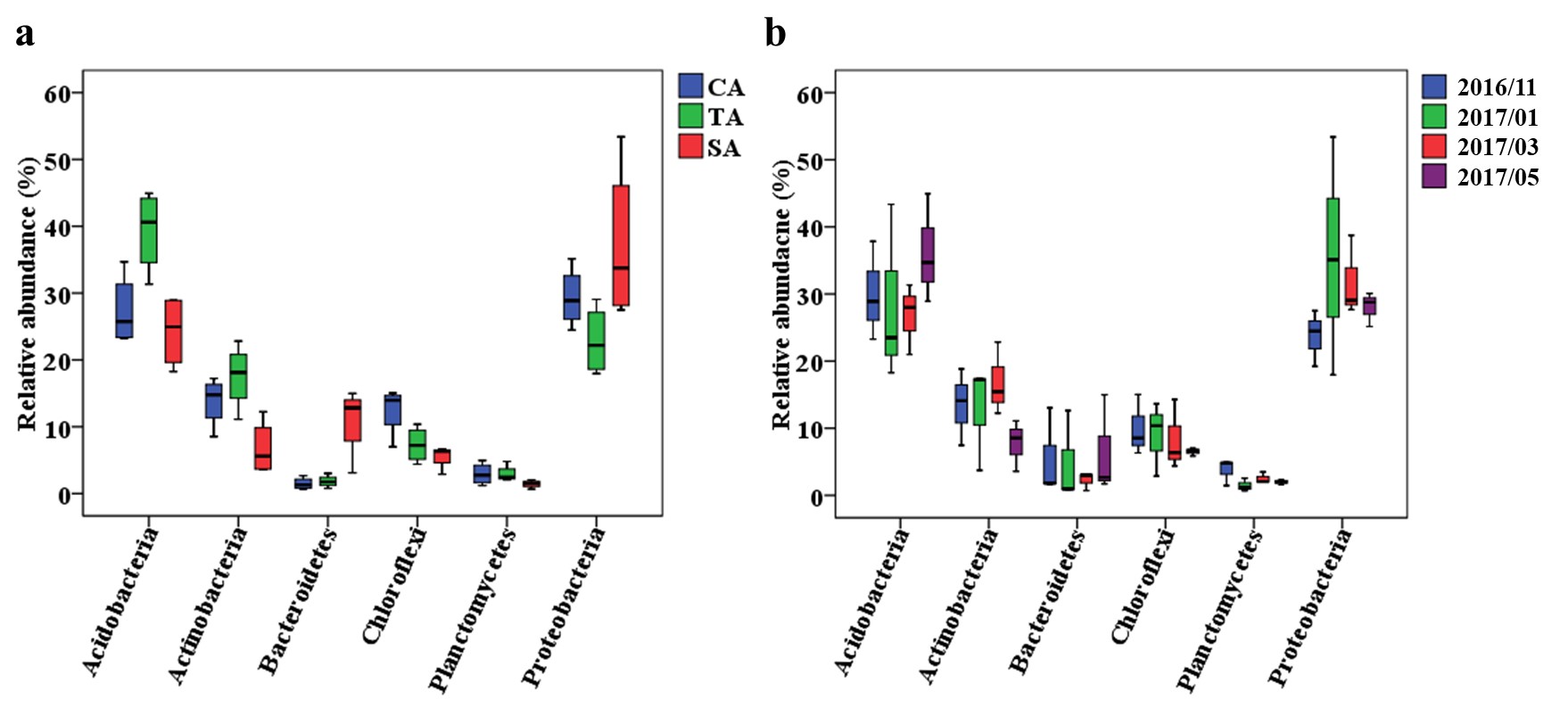


Fig. S3. Box plot showing (a) the distribution top six phyla at three soils and (b) at different sampling times.
