# Supplementary material for: Agricultural management practices influence the soil enzyme activity and bacterial community structure in tea plantations
Source: Bot Stud. 2021 May 18;62:8. doi: 10.1186/s40529-021-00314-9 (PMC8131499; doi:10.1186/s40529-021-00314-9)
Supplement: Supplementary file 6 — Additional file 6: Table S3. Modeling the enzymatic activity and bacterial community with soil chemical properties by multivariate linear regression ANOVA with the stepwise method. [file 40529_2021_314_MOESM6_ESM.docx]

Table S3. Modeling the enzymatic activity and bacterial community with soil chemical properties by multivariate linear regression ANOVA with the stepwise method.

| Equations | Model |  |
| --- | --- | --- |
| Acid phosphatase = –0.461+0.123 × organic matter | r^2^ = 0.676, F = 20.888, p-value = 0.001 | (1) |
| Arylsulfatase = –0.227+0.07 × pH | r^2^ = 0.963, F = 260.678, p-value = 0.000 | (2) |
| β-Glucosidase = –0.023 + 0.001 × K | r^2^ = 0.646, F = 18.276, p-value = 0.002 | (3) |
| SOBS = 26.327 + 301 × pH | r^2^ = 0.725, F = 26.423, p-value = 0.000 | (4) |
| Chao = 117.262 + 330.738 × pH | r^2^ = 0.761, F = 31.837, p-value = 0.000 | (5) |
| ACE = 181.788 + 317.184 × pH | r^2^ = 0.759, F = 31.486, p-value = 0.000 | (6) |
